# Supplementary material for: Modeling glioblastoma heterogeneity as a dynamic network of cell states
Source: Mol Syst Biol. 2021 Sep 16;17(9):e10105. doi: 10.15252/msb.202010105 (PMC8444284; doi:10.15252/msb.202010105)
Supplement: Supplementary file 5 — Source Data for Figure 3 [file MSB-17-e10105-s001.zip › Figure3A_sourcedata/GSEA_3065/hallmarks_state3.GseaPreranked.1623416274744/HALLMARK_CHOLESTEROL_HOMEOSTASIS.html]

Details for gene set HALLMARK\_CHOLESTEROL\_HOMEOSTASIS[GSEA]

|  || Dataset | state3 |
| Phenotype | NoPhenotypeAvailable |
| Upregulated in class | na\_pos |
| GeneSet | HALLMARK\_CHOLESTEROL\_HOMEOSTASIS |
| Enrichment Score (ES) | 0.5132023 |
| Normalized Enrichment Score (NES) | 1.8541478 |
| Nominal p-value | 0.0 |
| FDR q-value | 0.0012671778 |
| FWER p-Value | 0.003 |
Table: GSEA Results Summary

  

Fig 1: Enrichment plot: HALLMARK\_CHOLESTEROL\_HOMEOSTASIS      
 Profile of the Running ES Score & Positions of GeneSet Members on the Rank Ordered List

  

| PROBE | GENE SYMBOL | GENE\_TITLE | RANK IN GENE LIST | RANK METRIC SCORE | RUNNING ES | CORE ENRICHMENT || 1 | CD9 |  |  | 32 | 0.473 | 0.0472 | Yes |
| 2 | SQLE |  |  | 39 | 0.457 | 0.0957 | Yes |
| 3 | ALCAM |  |  | 45 | 0.440 | 0.1426 | Yes |
| 4 | LPL |  |  | 68 | 0.381 | 0.1810 | Yes |
| 5 | HMGCR |  |  | 77 | 0.369 | 0.2199 | Yes |
| 6 | SCD |  |  | 108 | 0.340 | 0.2530 | Yes |
| 7 | FADS2 |  |  | 153 | 0.309 | 0.2812 | Yes |
| 8 | HMGCS1 |  |  | 267 | 0.254 | 0.2955 | Yes |
| 9 | GUSB |  |  | 369 | 0.226 | 0.3083 | Yes |
| 10 | CTNNB1 |  |  | 373 | 0.224 | 0.3321 | Yes |
| 11 | TNFRSF12A |  |  | 380 | 0.223 | 0.3554 | Yes |
| 12 | LDLR |  |  | 498 | 0.199 | 0.3634 | Yes |
| 13 | FASN |  |  | 503 | 0.198 | 0.3843 | Yes |
| 14 | FDPS |  |  | 518 | 0.196 | 0.4038 | Yes |
| 15 | SREBF2 |  |  | 613 | 0.185 | 0.4129 | Yes |
| 16 | DHCR7 |  |  | 669 | 0.176 | 0.4255 | Yes |
| 17 | TMEM97 |  |  | 740 | 0.165 | 0.4353 | Yes |
| 18 | ANTXR2 |  |  | 871 | 0.151 | 0.4365 | Yes |
| 19 | EBP |  |  | 897 | 0.149 | 0.4497 | Yes |
| 20 | SC5D |  |  | 914 | 0.147 | 0.4637 | Yes |
| 21 | SEMA3B |  |  | 956 | 0.142 | 0.4743 | Yes |
| 22 | CYP51A1 |  |  | 976 | 0.140 | 0.4872 | Yes |
| 23 | CLU |  |  | 988 | 0.139 | 0.5009 | Yes |
| 24 | JAG1 |  |  | 1011 | 0.138 | 0.5132 | Yes |
| 25 | GPX8 |  |  | 1138 | 0.127 | 0.5124 | No |
| 26 | NSDHL |  |  | 1344 | 0.112 | 0.5009 | No |
| 27 | ATXN2 |  |  | 1366 | 0.110 | 0.5103 | No |
| 28 | LGMN |  |  | 1637 | 0.095 | 0.4895 | No |
| 29 | TM7SF2 |  |  | 2168 | 0.070 | 0.4361 | No |
| 30 | IDI1 |  |  | 2285 | 0.065 | 0.4297 | No |
| 31 | GLDC |  |  | 2660 | 0.051 | 0.3922 | No |
| 32 | ACAT2 |  |  | 2779 | 0.048 | 0.3837 | No |
| 33 | CHKA |  |  | 2947 | 0.043 | 0.3691 | No |
| 34 | CPEB2 |  |  | 3071 | 0.039 | 0.3592 | No |
| 35 | HSD17B7 |  |  | 3704 | 0.023 | 0.2889 | No |
| 36 | LSS |  |  | 3790 | 0.020 | 0.2813 | No |
| 37 | CXCL16 |  |  | 3810 | 0.020 | 0.2813 | No |
| 38 | ABCA2 |  |  | 3875 | 0.018 | 0.2758 | No |
| 39 | PLAUR |  |  | 4075 | 0.013 | 0.2544 | No |
| 40 | PLSCR1 |  |  | 4373 | 0.005 | 0.2207 | No |
| 41 | GNAI1 |  |  | 5194 | -0.016 | 0.1280 | No |
| 42 | MVD |  |  | 5311 | -0.019 | 0.1167 | No |
| 43 | STX5 |  |  | 5711 | -0.030 | 0.0741 | No |
| 44 | ACSS2 |  |  | 5962 | -0.038 | 0.0494 | No |
| 45 | ATF3 |  |  | 6159 | -0.045 | 0.0317 | No |
| 46 | ATF5 |  |  | 6238 | -0.048 | 0.0278 | No |
| 47 | MVK |  |  | 6273 | -0.049 | 0.0292 | No |
| 48 | ANXA5 |  |  | 6522 | -0.057 | 0.0068 | No |
| 49 | TP53INP1 |  |  | 6622 | -0.062 | 0.0021 | No |
| 50 | FDFT1 |  |  | 6637 | -0.063 | 0.0073 | No |
| 51 | ERRFI1 |  |  | 6678 | -0.065 | 0.0096 | No |
| 52 | PMVK |  |  | 7113 | -0.088 | -0.0308 | No |
| 53 | PCYT2 |  |  | 7140 | -0.090 | -0.0241 | No |
| 54 | ETHE1 |  |  | 7312 | -0.101 | -0.0329 | No |
| 55 | ECH1 |  |  | 7658 | -0.134 | -0.0581 | No |
| 56 | PNRC1 |  |  | 8133 | -0.212 | -0.0898 | No |
| 57 | ACTG1 |  |  | 8389 | -0.289 | -0.0879 | No |
| 58 | S100A11 |  |  | 8490 | -0.336 | -0.0633 | No |
| 59 | LGALS3 |  |  | 8522 | -0.347 | -0.0295 | No |
| 60 | FABP5 |  |  | 8699 | -0.518 | 0.0060 | No |
Table: GSEA details [plain text format]

  

Fig 2: HALLMARK\_CHOLESTEROL\_HOMEOSTASIS: Random ES distribution      
 Gene set null distribution of ES for **HALLMARK\_CHOLESTEROL\_HOMEOSTASIS**
